# Speed Constancy or Only Slowness: What Drives the Kappa Effect

**DOI:** 10.1371/journal.pone.0154013

**Published:** 2016-04-21

**Authors:** Youguo Chen, Bangwu Zhang, Konrad Paul Kording

**Affiliations:** 1 Key Laboratory of Cognition and Personality (Ministry of Education), Center of Studies for Psychology and Social Development, Faculty of psychology, Southwest University, Chongqing, China; 2 Department of Physical Medicine and Rehabilitation, Rehabilitation Institute of Chicago, Northwestern University, Chicago, Illinois, United States of America; Liaoning Normal University, CHINA

## Abstract

In the Kappa effect, two visual stimuli are given, and their spatial distance affects their perceived temporal interval. The classical model assumes constant speed while a competing Bayesian model assumes a slow speed prior. The two models are based on different assumptions about the statistical structure of the environment. Here we introduce a new visual experiment to distinguish between these models. When fit to the data, both the two models replicated human response, but the slowness model makes better behavioral predictions than the speed constancy model, and the estimated constant speed is close to the absolute threshold of speed. Our findings suggest that the Kappa effect appears to be due to slow speeds, and also modulated by spatial variance.

## Introduction

The Kappa effect is a spatiotemporal illusion where the perception of elapsed time between the sensory stimuli is systematically distorted by the irrelevant distance between the stimuli [1, 2]. In the simplest version, two lights are flashed with a chosen temporal interval at a given distance, and the perceived time increases with the spatial distance between the stimuli [3, 4]. In a more complicated scenario, three lights are flashed to define two successive temporal intervals and two adjoining spatial intervals. When the two temporal intervals are physically equal, the perceived time of longer spatial interval is longer than that of shorter spatial interval [1, 2].

A classical model explains the Kappa effect assuming constant speed within each trial [3, 5, 6]. It assumes that subjects have learned that objects in the environment move at constant speed [7], implicitly interpreting the static lights as moving [2, 4, 8]. This idea was captured by an algebraic model to quantitatively explain the Kappa effect [3, 5, 6]. It assumes that the perceived inter-stimulus time is a weighted average of the actual time and the expected time, calculated as the ratio of known distance and velocity. This model even explains an auditory (pitch) version of the Kappa effect [9, 10]. However, this weighted average model cannot tell us how the relative weights are determined.

Recently, a Bayesian model was proposed based on a slow velocity prior [11, 12]. It assumes an environment in which slower motions are more likely than faster ones, giving observers the expectation that objects move slowly [13–15]. The model combines this prior expectation with the observed spatiotemporal information into an optimal percept. The model does not only replicate the cutaneous rabbit illusion, the tactile Tau effect, the tactile Kappa effect, and other tactile spatiotemporal illusions, but it also explains tactile temporal order judgment and spatial attention effects [11, 12]. This raises the question if the model can also explain the visual Kappa effect.

In a typical Bayesian model, the mean of the posterior is a weighted average of the means of the prior and the likelihood [16, 17]; Similarly, in the classical model, the perceived time is the weighted average of the actual time and the expected time. This may suggest that the classical model, when properly defined, can also be written as a Bayesian model (also see [12]). Nothing prevents the assumption of constant speeds to be defined as a proper Bayesian prior.

Here we hypothesized that participants may expect constant speed and not just low speed. We formulate both the slow speed model and the classical (constant speed) model as a Bayesian model that deals with noisy data. We conducted a modified time reproduction task to replicate the simplest version of the Kappa effect [3, 4], and to understand which model best explains the Kappa effect.

## Experiment 1

### Methods

#### Participants

Nine right-handed participants (6 males and 3 females, 19 to 32 years of age) took part in the Experiment 1. Each participant had normal or corrected-to-normal visual acuity. Informed consent was obtained prior to participation. The project was approved by the review board of Southwest University, which were in accordance with the Declaration of Helsinki.

#### Stimuli and procedures

The visual stimuli were displayed on a black background in the center of a computer screen (screen resolution, 1024 × 768 pixels; refresh rate, 85 Hz). A white and a blue square were 2 mm in size (0.191°); a white circle was 6 mm in diameter (0.573°). We used MATLAB and Psychtoolbox to control stimulus presentation and record the subject responses [18]. The computer screen was placed about 60 cm in front of participants.

**Fig 1:**
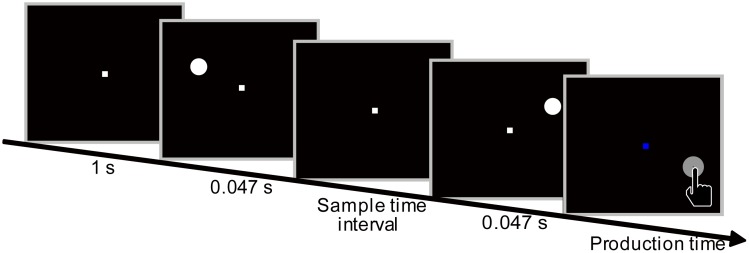
The illustration of one trial.

A modified time reproduction task was adopted to replicate the Kappa effect. The task was used to study visual Kappa effect [4] and an effect of temporal context on interval timing [19]. Given the Kappa effect was observed regardless of directions of circles flashed [1, 2, 8], we adopted one direction that circles flashed from left to right. At the beginning of each trial, a white square appeared in the center of screen for 1 s as a fixation target (Fig 1). Participants were instructed to fixate the central square throughout the trial. Two white circles flashed from the left to right visual fields in sequence, and then the central square turned blue. The vertical distance between the circles and the central square was 30 mm (2.865°). The horizontal distances between the two circles and the fixation square were equal. The distance between the two circles was randomly chosen from 17 distances (1.414°×i, i = 0, 1, 2…16). Participants were asked to estimate the sample time interval between the presentations of the two circles and to reproduce the time interval by pressing a key. The production times were measured from the onset of the blue fixation to the time participants responded.

We used two sample time intervals (0.8 s and 1.2 s). The two sample intervals were tested in separate blocks, the order of which was counterbalanced across participants. Each treatment consisted of 40 trials, so there were 1360 trials in the present study (2 × 17 × 40). Participants would have a short break (about half a minute) once they completed 170 trials, and they would have a rest for about 10 minutes after completing one sample time interval condition.

#### Bayesian models

Classical model. The classical model [6] assumes that observers tend to impute uniform motion to discontinuously displayed successive stimuli, their observed time is taken to be the weighted average of the given sample time interval (*t*_*s*_) and the expected time, *E*(*t*), that would be required to traverse the given distance (*l*) at constant speed (*v*_*0*_). The observed time can be written (S1 Appendix),

$$t_{e}=\omega t_{s}+\left(1-\omega \right)\frac{l}{v_{0}}$$ (1)

**Fig 2:**
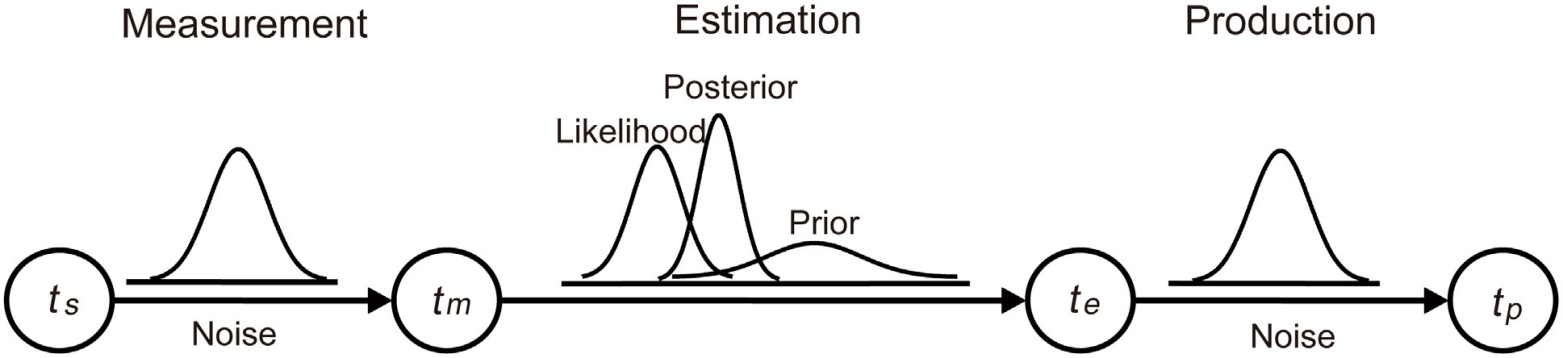
The three-stage model for time reproduction.

We continue to show that the same Eq 1 can be deduced from an appropriately defined Bayesian model. The Bayesian model uses the standard steps: A presented stimulus interval, a noisy measurement, a Bayesian estimation, and a production (Fig 2).

The three-stage Bayesian model was modified from an ideal observer model for time reproduction [19]. In the first stage, a sample interval (*t*_*s*_) is measured. The distribution of measured interval (*t*_*m*_) given *t*_*s*_ is *p*(*t*_*m*_|*t*_*s*_), which is a Gaussian distribution with mean *t*_*s*_ and standard deviation *σ*_*m*_. It is consistent with the scalar timing theory that internal representation of a temporal duration is a distribution of values which has an accurate mean [20].

The second stage is a Bayesian estimator. Participants have a prior belief of a constant speed (*v*_*0*_) on moving objects. Given moving distance (*l*), they believe a prior time interval distribution, *p*(*τ*), which is modeled as a Gaussian function with mean *l*/*v*_*0*_ and standard deviation *σ*_*τ*_. The prior distribution is:

$$p\left(\tau \right)=\frac{1}{\sqrt{2\pi }\sigma_{\tau }}e^{-\frac{{\left(\tau -\frac{l}{v_{0}}\right)}^{2}}{2\sigma_{\tau }^{2}}}$$ (2)

The likelihood is *p*(*t*_*m*_|*τ*), and the posterior distribution can be computed using Bayes rule.

$$p\left(\tau |t_{m}\right)\propto p\left(t_{m}|\tau \right)p\left(\tau \right)=\frac{1}{\sqrt{2\pi }\sigma_{m}}e^{-\frac{{\left(t_{m}-\tau \right)}^{2}}{2\sigma_{m}^{2}}}\cdot \frac{1}{\sqrt{2\pi }\sigma_{\tau }}e^{-\frac{{\left(\tau -\frac{l}{v_{0}}\right)}^{2}}{2\sigma_{\tau }^{2}}}$$ (3)

The mean of the posterior distribution is:

$$t_{e}=\frac{\sigma_{\tau }^{2}}{\sigma_{\tau }^{2}+\sigma_{m}^{2}}\cdot t_{m}+\frac{\sigma_{m}^{2}}{\sigma_{\tau }^{2}+\sigma_{m}^{2}}\cdot \frac{l}{v_{0}}$$ (4)

Given sample interval (*t*_*s*_), the estimated time (*t*_*e*_) is:

$$\langle t_{e}|t_{s}\rangle =\frac{\sigma_{\tau }^{2}}{\sigma_{\tau }^{2}+\sigma_{m}^{2}}\cdot t_{s}+\frac{\sigma_{m}^{2}}{\sigma_{\tau }^{2}+\sigma_{m}^{2}}\cdot \frac{l}{v_{0}}$$ (5)

Let $\omega =\frac{\sigma_{\tau }^{2}}{\sigma_{\tau }^{2}+\sigma_{m}^{2}}$

Thus,

$$\langle t_{e}|t_{s}\rangle =\omega \cdot t_{s}+\left(1-\omega \right)\cdot \frac{l}{v_{0}}$$ (6)

And we can see that our Eq 6, derived from a Bayesian constant speed model, is identical to Eq 1.

In the last stage, participants use *t*_*e*_ to produce *t*_*p*_. Given an estimated time *t*_*e*_, the distribution of production time (*t*_*p*_) was determined by *p*(*t*_*p*_|*t*_*e*_). Previous studies showed that the standard deviation of the production time increases linearly with their mean, a property that is termed scalar variability. Thus *σ*_*p*_ = *w*_*p*_*t*_*e*_, *w*_*p*_ was a Weber fraction [21–23],

$$p\left(t_{p}|t_{e}\right)=\frac{1}{\sqrt{2\pi }w_{p}t_{e}}e^{-\frac{{\left(t_{p}-t_{e}\right)}^{2}}{2{\left(w_{p}t_{e}\right)}^{2}}}$$ (7)

Slowness model. Goldreich [11] developed a Bayesian model to replicate the tactile Kappa effect and other tactile spatiotemporal illusions which is based on the assumption of slowness. The slow speed prior reflects observers’ expectation of slow movement, and it is modeled as a Gaussian function centered at zero [11, 12]. This model deduced a formula for the Kappa effect (S1 Appendix),

$$t_{s}=t_{e}(1-2{\left[\frac{l\left(\frac{\sigma_{t}}{\sigma_{s}}\right)\left(\frac{\sigma_{v}}{\sigma_{s}}\right)}{{\left(\frac{\sigma_{v}}{\sigma_{s}}t_{e}\right)}^{2}+2}\right]}^{2})$$ (8)

*l* was distance between two stimuli, *t*_*s*_ was sample time interval, *t*_*e*_ was estimated time, *σ*_*s*_ and *σ*_*t*_ were standard deviations (SD) of perceived spatial and temporal information, and *σ*_*v*_ was SD of prior speed. The estimated time (*t*_*e*_) can be computed using the fzero command in MATLAB (The MathWorks Inc.).

Finally, participants used *t*_*e*_ to produce *t*_*p*_, which was the same with the last stage of Bayesian version of the classical model.

#### Fitting the models to the data

We assumed that *t*_*p*_ values associated with any *t*_*s*_ were independent across trials. The joint conditional probability of individual *t*_*p*_ values across all N trials could be expressed as follows:

$$p\left(t_{p}^{1},t_{p}^{2},\ldots ,t_{p}^{N}|t_{e}\right)=\prod_{i=1}^{N}p(t_{p}^{i}|t_{e})$$ (9)

and

$$\text{log }p\left(t_{p}^{1},t_{p}^{2},\ldots ,t_{p}^{N}|t_{e}\right)=\sum_{i=1}^{N}\text{log }p(t_{p}^{i}|t_{e})$$ (10)

The parameters of the models were found by maximizing the likelihood using fminsearch command in MATLAB. For the classical model, previous study reported *ω* changes as a function of sample time intervals [6], thus four parameters were *ω*_*0*.*8*_ for the 0.8-s sample time interval condition, *ω*_*1*.*2*_ for the 1.2-s condition, *v*_*0*_ and *w*_*p*_.

For the slowness model, four parameters were *σ*_*t0*.*8*_ for the 0.8-s condition, *σ*_*t1*.*2*_ for the 1.2-s condition, *σ*_*v*_ and *w*_*p*_. The *σ*_*s*_ can be inferred according to previous studies [24–26]. In the constant method with a two-alternative forced choice paradigm, the just noticeable difference (JND) is defined as the difference between 25% and 75% on the psychometric functions, and then the standard deviation (*σ*) can be computed using JND for a Gaussian distribution, *σ* = *JND*/0.6475 [25, 26]. The visual acuity threshold is an application of JND in measuring spatial resolution of the visual processing system. A previous study reported two visual acuity thresholds (Vernier and grating resolutions) as functions of eccentricity were described by *k* = 0.93 × *ε*^0.69^ and *k* = 1.34 × *ε*^0.71^ respectively (the unit of *κ* is minute not degree), where *κ* is the visual acuity threshold (or JND of spatial resolution), and *ε* is the eccentricity [24]. Thus the *σ*_*s*_ was written as functions of the eccentricity (the unit of *σ*_*s*_ is degree not minute), *σ*_*s*_ = 0.0239 × *ε*^0.69^ (Vernier resolution) and *σ*_*s*_ = 0.0345 × *ε*^0.71^ (grating resolution). To evaluate the effect of different types of visual acuity thresholds on model fitting, the Vernier and grating resolutions were used to compute *σ*_*s*_ respectively.

Akaike information criterion (AIC) was adopted to evaluate the goodness of model fitting. AIC difference (Δ) was computed for competitive models. Δ is the difference of AIC values between each model and the best model (smallest AIC) (Δ = AIC_i_-AIC_min_) [27, 28].

## Results

Here we wanted to ask if the Kappa effect could be best explained assuming constant speeds or by assuming slowness. We employed a modified time reproduction task to analyze the simplest version of the Kappa effect (Fig 1). Two circles were presented in sequence to define two temporal and 17 spatial intervals. This allows us to characterize the reproduced interval as a function of distance and temporal delay.

**Fig 3:**
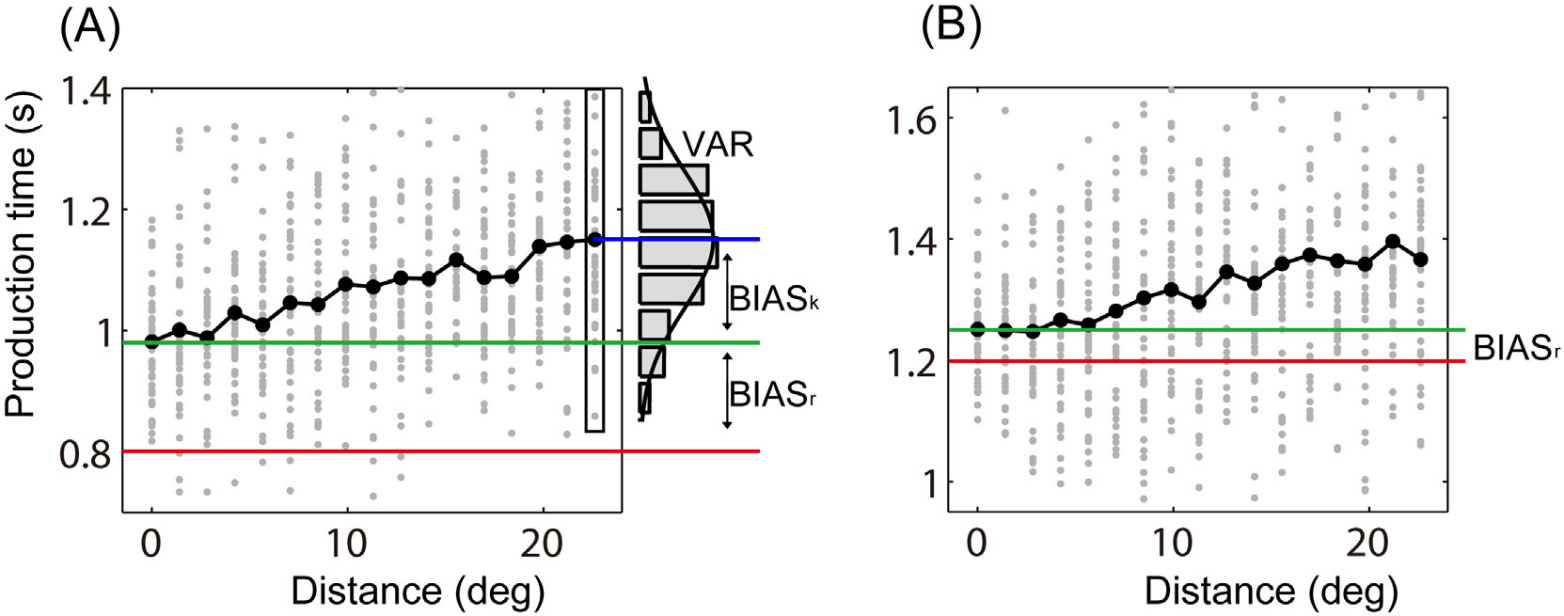
The production time for a typical subject. (A) The 0.8-s sample time interval condition. (B) The 1.2-s condition. Gray dots stand for production time for every trial. Black spots connected with black lines show the mean for each distance condition. Red lines indicate the values of sample time intervals, green lines indicate the production time when the two targets are at the same location, and blue lines indicate mean production time of a treatment.

As a baseline for all tests we need to know the response bias (BIAS_r_) when the two targets are at the same position. For each sample time interval, we computed a mean production time when the two targets were right above the fixation (the vertical distance was 30 mm), and then the BIAS_r_ was obtained by subtracting the sample time interval from the mean production time (Fig 3). One sample *t*-test revealed that the mean BIAS_r_ was significant higher than zero for the 0.8-s condition [mean ± SE: 0.137 ± 0.045 s, *t*(8) = 3.016, *p* < 0.05, Cohen’s *d* = 1.007], and it was not significant for the 1.2-s condition [0.045 ± 0.066 s, *t*(8) = 0.681, *p* > 0.05, Cohen’s *d* = 0.227]. We corrected all response biases by subtracting the measured subject specific response bias, despite the fact that these biases are quite small.

**Fig 4:**
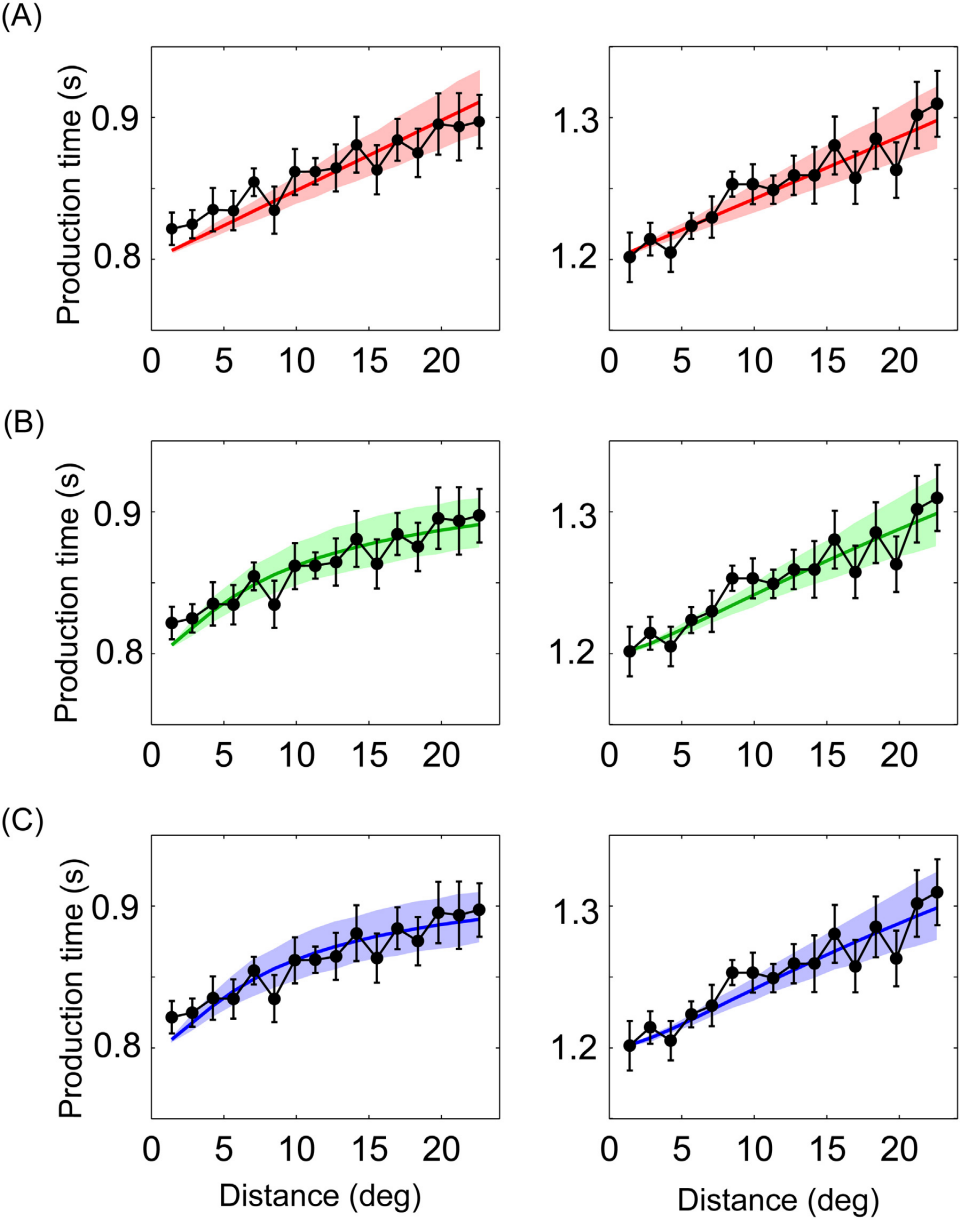
The production time for all participants and the Bayesian models. Left: the 0.8-s sample time interval condition; Right: the 1.2-s condition. (A) The classical model. (B) The slowness model with the Vernier resolution. (C) The slowness model with the grating resolution. Black spots connected with black lines show the mean response of all participants. Red, green and blue lines represent the mean response of the Bayesian models. The error bar and shadow indicate one standard error.

For each treatment, BIAS_k_ is the difference of the mean production time between the treatment and the baseline when the two targets are at same location, and VAR is the corresponding variance (Fig 3). BIAS_k_ and VAR were obtained from single-subject data. The BIAS_k_ increased with increasing distance between the two circles (Figs 3 and 4), which is consistent with previous studies on the Kappa effect [4, 6, 29]. For all participants, the mean VAR was significantly smaller in the 0.8-s sample interval condition (mean ± SE: 0.027 ± 0.005 s^2^) than that in the 1.2-s condition (0.051 ± 0.014 s^2^) [*t*(8) = -2.57, *p* < 0.05, Cohen’s *d* = -0.862]. Reproduction of longer sample time intervals accompanying more uncertainty is in line with scalar variability [21–23].

When fit to the data, both the classical model and the slowness model replicated human results (Fig 4). The classical model predicts production time as a linear function of distance. Its parameters seem intuitive, with two *ω* very close to one, a *v*_*0*_ of 0.2°/s, and a *w*_*p*_ of 0.2 (Table 1).

**Table 1:** Best-fitting parameter values of the classical model for each of nine participants.

	*ω*_0.8_	*ω*_1.2_	*v*_*0*_	*w*_*p*_
S1	0.9983	0.9986	0.2290	0.1501
S2	0.9989	0.9993	0.1851	0.1652
S3	0.9995	0.9986	0.2107	0.1400
S4	0.9979	0.9999	0.2161	0.1266
S5	0.9990	0.9979	0.2139	0.1295
S6	0.9996	0.9994	0.1752	0.1246
S7	0.9984	0.9995	0.2065	0.2581
S8	0.9999	0.9987	0.2638	0.2657
S9	0.9990	0.9994	0.2764	0.2594
Mean	0.9989	0.9990	0.2196	0.1799

For the slowness model, the estimated time increases more slowly with long distance than that with short distance in the 0.8-s condition, but it is close to the response of the classical model in the 1.2-s condition. The slowness model with two different visual acuity thresholds produce almost the same estimated time (Fig 4B and 4C), and their best-fitting parameter values are approximately equal (Table 2). *σ*_*t*_ is about 0.01 s and 0.02 s for the 0.8-s and 1.2-s conditions; *σ*_*v*_ and *w*_*p*_ are about 0.9°/s and 0.2, respectively. The slowness model appears better to qualitatively explain the data (Fig 4).

**Table 2:** Best-fitting parameter values of the slowness model with the Vernier (SMV) and grating (SMG) resolutions for each of nine participants.

	SMV				SMG			
	*σ*_*t0*.*8*_	*σ*_*t1*.*2*_	*σ*_*v*_	*w*_*p*_	*σ*_*t0*.*8*_	*σ*_*t1*.*2*_	*σ*_*v*_	*w*_*p*_
S1	0.0068	0.0076	0.2531	0.1499	0.0107	0.0121	0.4085	0.1499
S2	0.0060	0.0056	0.2210	0.1650	0.0094	0.0089	0.3595	0.1650
S3	0.0043	0.0077	0.1616	0.1394	0.0066	0.0121	0.2626	0.1394
S4	0.0081	0.0022	0.1701	0.1248	0.0127	0.0034	0.2779	0.1249
S5	0.0181	0.0510	1.8804	0.1299	0.0235	0.0657	2.4121	0.1299
S6	0.0170	0.0355	2.5765	0.1248	0.0180	0.0372	2.6780	0.1248
S7	0.0079	0.0048	0.1343	0.2556	0.0122	0.0075	0.2220	0.2556
S8	0.0052	0.0380	2.1775	0.2659	0.0046	0.0327	1.8416	0.2659
S9	0.0098	0.0065	0.0580	0.2566	0.0137	0.0093	0.1043	0.2567
Mean	0.0092	0.0177	0.8481	0.1791	0.0124	0.0210	0.9518	0.1791

AIC difference (Δ) was obtained from each participant to evaluate the goodness of model fitting (Table 3). The larger Δ is, the less plausible it is that the fitted model. According to the levels of empirical support for model (0 ≤ Δ ≤ 2, substantial; 4 ≤ Δ ≤ 7, considerably less; Δ > 10, essentially none) [27], the slowness model is superior for 6 out of 9 participants in the 0.8-s condition, and 1 out of 9 participants in the 1.2-s condition (Δ ≥ 4). Sign test revealed that AIC of the classical model was significant larger than that of the slowness model for the 0.8-s condition (*p* < 0.01), whereas the difference of AIC between two models was not significant for the 1.2-s condition (*p* > 0.05).

**Table 3:** Model comparison for each of nine participants.

	0.8-s condition						1.2-s condition					
	CM		SMV		SMG		CM		SMV		SMG	
	AIC	Δ	AIC	Δ	AIC	Δ	AIC	Δ	AIC	Δ	AIC	Δ
S1	-707	1	-708	0	-708	0	-334	2	-336	0	-336	0
S2	-555	1	-556	0	-556	0	-306	0	-306	0	-305	1
S3	-905	4	-909	0	-908	1	-392	1	-393	0	-393	0
S4	-650	16	-666	0	-666	0	-863	8	-871	0	-871	0
S5	-755	4	-759	0	-759	0	-680	1	-681	0	-681	0
S6	-908	6	-915	0	-914	1	-728	0	-727	1	-727	1
S7	-119	7	-126	0	-126	0	406	0	406	0	406	0
S8	-205	1	-206	0	-205	1	469	2	467	0	467	0
S9	-64	7	-71	0	-71	0	293	1	292	0	292	0

CM represents the classical model, SMV represents the slowness model with the Vernier resolution, and SMG represents the slowness model with the grating resolution.

**Fig 5:**
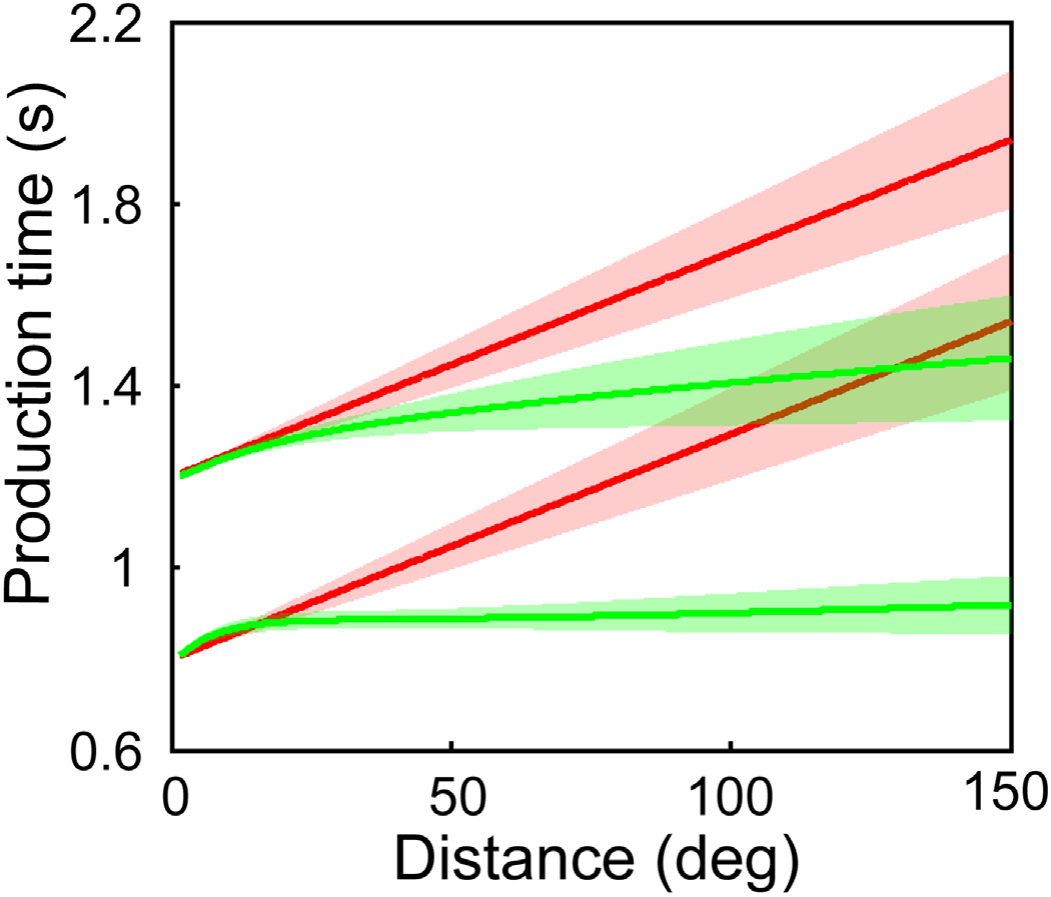
Predictions of the classical (red) and the slowness (green) models. Bottom: the 0.8-s sample time interval condition; Top: the 1.2-s condition. The shadow indicates one standard error.

Predictions of the two models were plotted with distances between two circles from 1.4° to 150° based on the best-fitting parameters (Fig 5). Only the Vernier resolution was used, because of the identical responses for the slowness model with two visual acuity thresholds (Fig 4). Production time increases linearly with increase of distance for the classical model; it increases as increasing distance but with a tendency of deceleration for the slowness model. The difference between two models increases with increasing distance. It indicates that a much larger range of distance should be used in the future work on Kappa effect.

## Experiment 2

A basic hypothesis in the Experiment 1 is that, there is only one response bias for one sample time of a participant. We designed a supplementary experiment to test if bias was modulated by location.

### Method

We recruited an extra nine right-handed participants (5 males and 4 females, 19 to 29 years of age) took part in the Experiment 2. These participants did not take part in the Experiment 1. Informed consent was obtained prior to participation. The project was approved by the review board of Southwest University, which were in accordance with the Declaration of Helsinki.

The visual stimuli and apparatus were identical with the Experiment 1. Two white circles flashed at the same location in each trial. The vertical distance between the circles and the central square was 30 mm (2.865°). The horizontal distances between the circles and the fixation square were 12° in the left visual field, 0°, and 12° in the right. There were two sample time intervals (0.8 s and 1.2 s). Two sample time intervals and three locations were randomly presented. Each treatment consisted of 40 trials, so there were 240 trials in the Experiment 2 (2 × 3 × 40). The rest were identical with the Experiment 2.

### Result

The response biases (BIAS_r_) were obtained for each sample time interval each location of nine participants (Table 4). Two-way repeated measurement ANOVA was conducted on the BIAS_r_ with Duration (0.8 s and 1.2 s) and Location (12° in the left, 0° and 12° in the right) as within subject factors. The main effects of Duration [*F*(1, 8) = 3.586, *p* = 0.095, *η*_p_^2^ = 0.310] and Location [*F*(2, 16) = 0.150, *p* > 0.05, *η*_p_^2^ = 0.018] were not significant, and the interaction of Duration × Location was not significant [*F*(2, 16) = 0.137, *p* > 0.05, *η*_p_^2^ = 0.017]. The results suggest that the BIAS_r_ was not significantly modulated by location.

**Table 4:** Mean and standard error of response biases in the left, central and right visual fields for nine participants.

	Left	Central	Right
0.8-s			
M	0.134	0.133	0.146
SE	0.056	0.038	0.059
1.2-s			
M	0.047	0.054	0.057
SE	0.042	0.028	0.042

## General Discussion

Here we repeated a typical Kappa effect that the production time increases with the distance between two circles. Then we compared the fits of the Bayesian models between assuming constant speeds and slowness. Both the classical model and the slowness model replicated human response (Fig 4). AIC index provided quantitative evidence that the slowness model fits data better than the classical model.

The response bias was found in the present study, which is consistent with previous study [30–32]. As early as 1868, Vierordt [33] discovered that short durations are reproduced longer than the standard durations, and long durations shorter (Vierordt’s law). To the best of our knowledge, no study reported whether the response bias is modulated by locations of visual stimuli, thus we hypothesized that there is only one response bias for one sample time interval of a participant (Fig 3). We conducted a supplementary experiment to answer whether the response biases at the peripheral locations are the same to that at the central locations (Experiment 2). The difference of response bias between the central and the peripheral areas was not significant, which suggests that the response bias is not modulated by the locations of visual stimuli.

It is necessary to interpret a detail about response bias in the classical model (S1 Appendix). Jones and Huang [3, 6] did not indicate the response bias explicitly. They proposed, given a temporal duration, the internal code of observed time is the combination of the given duration’s scale value and the expected time for Kappa effect (Equation A2 in S1 Appendix). When they fitted the equation to the data, the scale value was contributed by the sample time and the response bias (Equation A4 in S1 Appendix) [6]. Thus the term ‘scale value’ has an ambiguous psychological meaning. We separated response bias from the sample time, that is, the production time is the sum of Kappa effect and response bias (Equation A5 in S1 Appendix). The separation interpretation is equivalent to Jones and Huang’s equation mathematically, but has an unambiguous psychological meaning.

We can fit models to the data in two ways. The first way is to correct response bias before data fitting. An advantage is that we can focus on the Kappa effect and avoid the influence of response bias during data fitting. The second way is to fit the data without correcting, and consider the response bias as a parameter. A disadvantage is that overfitting may occur when a model has too many parameters. We did a supplementary analysis for the two models. For the classical model, six parameters were *ω*_0.8_, *ω*_1.2_, *v*_0_, *w*_p_, BIAS_r0.8_ and BIAS_r1.2_ (Equation A5 in S1 Appendix). The *ω* should smaller than one, but the *ω* was larger than/equal to 1 for four participants (S4, S5, S7 and S9). For the slowness model, the mean of *tp* is modeled as a Gaussian function centered at *t*_*e*_+BIAS_r_. Six parameters are *σ*_t0.8_, *σ*_t1.2_, *σ*_*v*_, *w*_*p*_, BIAS_r0.8_ and BIAS_r1.2_. The *σ*_t_ and *σ*_*v*_ should be positive, but they are negative or zero for some participants (Vernier acuity: S8; grating acuity: S2, S4, S8 and S9). The overfitting might occur for those participants with unreasonable parameters. For other participants, parameters were close to the fitted values with response bias corrected (Tables 1 and 2). This analysis large convinced us that our analysis is solid.

The classical model and the slowness model both assume a speed prior but with different statistic structures. The slowness model is based on a low speed prior, which assumes that the prior for motion is centered on zero [13–15, 34, 35]. The classical model assumes that the perceived inter-stimulus time is a weighted average of the actual time and the expected time, calculated as the ratio of known distance and prior speed. Logically, a constant speed can be slow. Estimated time is longer than physical time between two circles in the Kappa effect. Given the mean of posterior is larger than the mean of likelihood, the mean of prior time interval must be larger than that of likelihood (Fig 2), in other words, the prior speed must be slower than speeds of circles flashed (*v* = *l*/*t*). Consistent with prediction of the classical model (Fig 2), we found that the constant speed is about 0.2°/s, which is far less than the speeds of circles flashed in the present study (1.2 to 28.3°/s). A previous study reported that the absolute threshold of speed is 0.12°/s for old participant group, and 0.09°/s for young participant group [36]. In that the constant speed is close to the absolute threshold of the speed, it is reasonable to consider the constant speed as slowness, which is the reason that both the classical model and the slowness model replicated human response (Fig 4).

The slow speed prior, which was widely studied in the field of motion perception [37], was proven to mirror statistical structures of the visual environment that fast-moving objects are relatively rare [38]. The neural substrate of the slow speed prior remains under debate. Vintch and Gardner [39] suggested that the slow speed prior is encoded by neural populations in the same early cortical areas that provide sensory evidence (V1 area). Jogan and Stocker [40] indicated the combination of the sensory information with the prior belief is likely to occur downstream of medial temporal area (MT), however, it does not imply that the prior information is also represented in the area MT as has been proposed [41]. More conclusive evidence is needed to identify the neural substrate of the slow speed prior.

Both the classical model and the slowness model employ the slow speed prior, however, the slowness model is superior to the classical model in data fitting. There seems only a tendency of deceleration for production time with increasing distance in the 0.8-s condition rather than the 1.2-s condition (Fig 4). We believe that this is because that the distance is not long enough for the 1.2 s condition in the present study. We computed the predictions of the two models with distance from 1.4° to 150° based on the best-fitting parameters. The slowness model predicts a tendency of deceleration for production time in the 0.8-s and 1.2-s conditions (Fig 5). Thus, the production time from 1.4° to 22.6° in the 1.2-s condition, seems like a straight line, but is really part of a curve. The main difference between two models is that the slowness model considers spatial variance as an influence of the Kappa effect, but the classical model does not. In the visual system, visual acuity reduces sharply with increase of eccentricity [24]. The Vernier and the grating resolutions were used to compute spatial variance for each distance in fitting the slowness model, which indicates that spatial variance increases with increase of eccentricity. Furthermore, it was proven that, the same physical distance with a larger spatial variance seems shorter [11], and then a shorter length perception leads to a shorter duration perception. Therefore, the slowness model predicted a tendency of deceleration for production time with increasing distance, which is the reason that the slowness model fits data better than the classical model (Fig 4).

Both the classical and the slowness models have advantages and disadvantages. An advantage of the classical model is that, it is based on a simple idea that the perceived time is a weighted average of a given time and the expected time [6]. Here we revealed that the relative weight was determined by uncertainty of measurement and prior time interval. It is consistent with previous studies that the central nervous system (CNS) incorporates knowledge about temporal uncertainty of internal and the environment to produce an optimal timing [19, 42, 43]. The classical model also has disadvantages. First, the linear classical model fitted data worse than the nonlinear slowness model in the 0.8-s condition. Some nonlinear features of spatial information (e.g. spatial variance) should be combined into the model. Second, the relative weight *ω* changes as a function of sample time intervals [6], which prevents the model from predicting production time for a new sample time with the best-fitting parameters obtained.

The slowness model is based on probability distributions of neural activity [11]. An advantage of this model is that the uncertainties of spatiotemporal information were considered. It predicts a tendency of deceleration for production time (Figs 4 and 5), which enables the model to fit data better than the linear model. The first disadvantage of the slowness model is its complex expression. The estimated time cannot be written as a function of sample time for the model, *t*_*e*_ = *f*(*t*_*s*_). We cannot obtain a precise estimated time, but only an approximate one by a numerical method. Second, approximate spatial variances were inferred from previous studies [24–26]. The spatial variances remained unchanged for nine participants. Individual differences, luminance contrast, and shapes of visual stimuli may modulate the spatial variances. Third, the uncertainty of temporal information changed with sample time (Table 2). Given best-fitting parameters, the change of parameter prevents the model from predicting production time for a new sample time. To develop a better model of Kappa effect, the advantages and disadvantages of the two models should be considered.

In summary, we conducted a new experiment to explore the cognitive mechanism underlying the Kappa effect. Both the classical and the slowness models replicated human response, because the two models are both based on the slow speed prior. The slowness model further considers spatial variance as an influence of the Kappa effect, which is the reason why the slowness model fits data better than the classical model. To improve the classical model, it is necessary to integrate the spatial variance into the model in the future work.

## Supporting Information

S1 Appendix Details of the classical and the slowness models. (PDF)

S1 Data Raw data set of Experiment 1. (XLS)

S2 Data Raw data set of Experiment 2. (XLS)
