# Supplementary material for: Speed Constancy or Only Slowness: What Drives the Kappa Effect
Source: PLoS One. 2016 Apr 21;11(4):e0154013. doi: 10.1371/journal.pone.0154013 (PMC4839579; doi:10.1371/journal.pone.0154013)
Supplement: S1 Appendix — (PDF) [file pone.0154013.s001.pdf]

Appendix

Classical model

Given that two lights are flashed with a chosen temporal interval (t_s) at a given distance (l), the Kappa effect can be disassembled into three steps [1].

First, the sample time interval is transferred to a scale value (t_s^*) according to a psychophysical function Φ_t. It can be written,

$$t_s^* = \phi_t(t_s) \qquad \text{(A1)}$$

Second, an internal code (Ψ_t) of observed time can be written as a weighted combination of the scale value t_s^* and the expected time,

$$\psi_t = \omega t_s^* + (1 - \omega)E(t) \qquad \text{(A2)}$$

where $E(t) = \frac{l}{v_0}$, and v_0 is a constant speed.

Finally, the observed response t_p is produced based on the internal code ψ_t and a psychomotor function h_t which maps ψ_t into the observed response.

$$t_p = h_t(\psi_t) \qquad \text{(A3)}$$

Jones and Huang [1] indicated that the psychophysical function (Φ_t) was linear. Given the sample time interval t_s, the scale value can be written as a sum of t_s and a deviant δ.

Let $t_s^* = t_s + \delta$,

Thus, $\psi_t = \omega(t_s + \delta) + (1 - \omega)\frac{l}{v_0}$ (A4)

We define $BIAS_r = \omega\delta$ which indicates the response bias. Equation A4 is equivalent to,

$$\psi_t = \omega t_s + (1 - \omega)\frac{l}{v_0} + BIAS_r \quad \text{(A5)}$$

This hypothesis is consistent with previous findings that time intervals below about 3 s are produced slightly longer, whereas intervals above about 3 s are produced systematically shorter in the temporal reproduction task [2-5]. We can correct BIAS_r for data, and fit a simpler model,

$$t_e = \omega t_s + (1 - \omega)\frac{l}{v_0} \quad \text{(A6)}$$

Slowness model

The slowness model is based on probability distribution of neural activities [6]. A tactile stimulus, such as a tap to a particular location on the skin, will evoke a neural response D. Given that location of the first tap is x_1, the probability of this neural activity can be modeled as a Gaussian function of space x, and centered at the true tap position x_1,

$$p(D|x) \propto \frac{1}{\sigma_s} e^{-\frac{(x-x_1)^2}{2\sigma_s^2}} \quad \text{(A7)}$$

Given that the second tap location is x_2, the speed and the time interval between two taps is v and τ respectively, the probability of neural activity for the second tap can be written,

$$p(D|x,v,\tau) \propto \frac{1}{\sigma_s} e^{-\frac{(x+v\tau-x_2)^2}{2\sigma_s^2}} \qquad \text{(A8)}$$

The neural activity also can be modeled as a Gaussian function of time t, and centered at the true tap time. The actual time of the first tap is defined as zero, and that of the second tap, as t_s. The neural activities of two taps are,

$$p(D|t) \propto \frac{1}{\sigma_t} e^{-\frac{t^2}{2\sigma_t^2}} \qquad \text{(A9)}$$

$$p(D|t,\tau) \propto \frac{1}{\sigma_t} e^{-\frac{(t+\tau-t_s)^2}{2\sigma_t^2}} \qquad \text{(A10)}$$

The likelihood of neural activity during the trajectory (two taps) is the product of the probabilities in space and time.

$$p(D|x,v,t,\tau) \propto \frac{1}{\sigma_s^2\sigma_t^2} e^{-\left[\frac{(x-x_1)^2+(x+v\tau-x_2)^2}{2\sigma_s^2}+\frac{t^2+(t+\tau-t_s)^2}{2\sigma_t^2}\right]} \qquad \text{(A11)}$$

The slow speed prior reflects an expectation for slow movement, and it is modeled as a Gaussian function centered at zero,

$$p(x,v,t,\tau) \propto \frac{1}{\sigma_v} e^{-\frac{v^2}{2\sigma_v^2}} \qquad \text{(A12)}$$

Then the posterior distribution can be computed using Bayes rule,

$$p(x,v,t,\tau|D) \propto p(D|x,v,t,\tau)p(x,v,t,\tau)$$

$$\propto \frac{1}{\sigma_s^2 \sigma_t^2 \sigma_v} e^{-\left[\frac{(x-x_1)^2+(x+v\tau-x_2)^2}{2\sigma_s^2}+\frac{t^2+(t+\tau-t_s)^2}{2\sigma_t^2}+\frac{v^2}{2\sigma_v^2}\right]} \quad \text{(A13)}$$

The mode of the posterior can be obtained by setting to zero the partial derivatives of the exponent of Equation A13 with respect to *x*, *v*, *t* and τ. The perceived time t_e is the value of τ at the mode of the posterior, and the equation relating t_s to t_e is,

$$t_s = t_e(1 - 2\left[\frac{l\left(\frac{\sigma_t}{\sigma_s}\right)\left(\frac{\sigma_v}{\sigma_s}\right)}{\left(\frac{\sigma_v}{\sigma_s}t_e\right)^2+2}\right]^2) \quad \text{(A14)}$$

References

1. Jones B, Huang YL. Space-time dependencies in psychophysical judgment of extent and duration: Algebraic models of the tau and kappa effects. Psychological Bulletin. 1982;91(1):128-42. doi: doi: 10.1037/0033-2909.91.1.128.

2. Pöppel E. A hierarchical model of temporal perception Trends in Cognitive Sciences. 1997;1(2):56-61. doi: 10.1016/S1364-6613(97)01008-5. pmid: 21223864.

3. Szelag E, Kowalska J, Rymarczyk K, Poppel E. Duration processing in children as determined by time reproduction: implications for a few seconds temporal window. Acta Psychologica. 2002;110(1):1-19. doi: 10.1016/S0001-6918(01)00067-1. pmid: 12005225.

4. Ulbrich P, Churan J, Fink M, Wittmann M. Temporal reproduction: Further evidence for two processes. Acta Psychologica. 2007;125(1):51-65. doi: 10.1016/j.actpsy.2006.06.004. pmid: 16904621.

5. Kagerer FA, Wittmann M, Szelag E, Von Steinbuchel N. Cortical involvement in temporal reproduction: evidence for differential roles of the hemispheres. Neuropsychologia. 2002;40(3):357-66. doi: 10.1016/S0028-3932(01)00111-7. pmid: 11684169.

6. Goldreich D. A Bayesian perceptual model replicates the cutaneous rabbit and other tactile spatiotemporal illusions. PLoS ONE. 2007;2(3):e333. doi: 10.1371/journal.pone.0000333. pmid: 17389923.
